# Practice variation and practice guidelines: Attitudes of generalist and specialist physicians, nurse practitioners, and physician assistants

**DOI:** 10.1371/journal.pone.0191943

**Published:** 2018-01-31

**Authors:** David A. Cook, Laurie J. Pencille, Denise M. Dupras, Jane A. Linderbaum, V. Shane Pankratz, John M. Wilkinson

**Affiliations:** 1 Knowledge Delivery Center, Mayo Clinic, Rochester, Minnesota, United States of America; 2 Mayo Clinic Online Learning, Mayo Clinic College of Medicine, Rochester, Minnesota, United States of America; 3 Division of General Internal Medicine, Mayo Clinic, Rochester, Minnesota, United States of America; 4 Center for the Science of Health Care Delivery, Mayo Clinic, Rochester, Minnesota, United States of America; 5 Division of Primary Care Internal Medicine, Mayo Clinic, Rochester, Minnesota, United States of America; 6 Department of Cardiovascular Diseases, Mayo Clinic, Rochester, Minnesota, United States of America; 7 Department of Internal Medicine, University of New Mexico Health Sciences Center, Albuquerque, New Mexico, United States of America; 8 Department of Family Medicine, Mayo Clinic, Rochester, Minnesota, United States of America; Dartmouth-Hitchcock Medical Center, UNITED STATES

## Abstract

**Objective:**

To understand clinicians' beliefs about practice variation and how variation might be reduced.

**Methods:**

We surveyed board-certified physicians (N = 178), nurse practitioners (N = 60), and physician assistants (N = 12) at an academic medical center and two community clinics, representing family medicine, general internal medicine, and cardiology, from February—April 2016. The Internet-based questionnaire ascertained clinicians' beliefs regarding practice variation, clinical practice guidelines, and costs.

**Results:**

Respondents agreed that practice variation should be reduced (mean [SD] 4.5 [1.1]; 1 = strongly disagree, 6 = strongly agree), but agreed less strongly (4.1 [1.0]) that it can realistically be reduced. They moderately agreed that variation is justified by situational differences (3.9 [1.2]). They strongly agreed (5.2 [0.8]) that clinicians should help reduce healthcare costs, but agreed less strongly (4.4 [1.1]) that reducing practice variation would reduce costs. Nearly all respondents (234/249 [94%]) currently depend on practice guidelines. Clinicians rated differences in clinician style and experience as most influencing practice variation, and inaccessibility of guidelines as least influential. Time to apply standards, and patient decision aids, were rated most likely to help standardize practice. Nurse practitioners and physicians assistants (vs physicians) and less experienced (vs senior) clinicians rated more favorably several factors that might help to standardize practice. Differences by specialty and academic vs community practice were small.

**Conclusions:**

Clinicians believe that practice variation should be reduced, but are less certain that this can be achieved. Accessibility of guidelines is not a significant barrier to practice standardization, whereas more time to apply standards is viewed as potentially helpful.

## Introduction

Practice variation is an important target for clinical systems improvement. Although some variation in clinical practice is justified, unwarranted variation–"variation that is not explained on the basis of illness, patient risk factors or patient preferences"[1]–is common. This can lead to underuse of effective care, overuse of non-beneficial services, and emphasis on physician opinions rather than patient preferences.[2, 3] Unwarranted variation has been linked to suboptimal outcomes[4–8] and to increased cost for the same outcome (i.e., inefficient care).[9, 10]

While clinical practice guidelines offer one potential solution to unwarranted practice variation,[5, 11, 12] implementation of guidelines encounters resistance, including both unintentional and intentional non-adherence.[13–17] Studies exploring clinicians' reasons for resistance to guidelines have identified concerns about decreased autonomy, oversimplification of medicine, uncertainty regarding the evidence base, financial conflicts of interest, and potential litigation.[18–23] Interventions to improve guideline adherence have met with only modest success.[24] Other approaches to reducing practice variation include patient decision aids,[25, 26] clinical decision support,[27–29] provider performance feedback,[29, 30] provider financial incentives,[31–33] and regulatory changes,[34] yet these too have had limited impact. Given these suboptimal results, we believe that before further trying to *solve* the problem of practice variation, we ought to better *understand* the nature of practice variation itself, including clinician beliefs about the issue.

Qualitative studies have empirically identified sources of practice variation, including the clinician (including personal experiences and attitudes), the patient and family, and the work environment.[35–37] Conceptual models have added social values and regulatory bodies to the list.[38] Yet we found no quantitative studies that directly explored the prevalence of clinician attitudes about practice variation. Moreover, studies exploring attitudes about clinical practice guidelines[13, 19, 20] and evidence-based medicine[39, 40] have incompletely explored how attitudes vary across clinician characteristics such as time in practice, provider type, and specialty. To address these gaps, we surveyed generalist and specialist clinicians to answer the following research questions:

1. What do clinicians believe about the sources and impact of practice variation?
2. What do clinicians believe about the credibility and utility of clinical guidelines and other approaches to reduce variation?
3. How do these beliefs vary by provider type, time in practice, specialty, and practice location?

We anticipated that nurse practitioners / physician assistants (given the pragmatic nature of their training and practice), clinicians early in their career (who may rely on external supports as they continue to learn), and generalists (who must treat a wide variety of often-unfamiliar conditions), would have more favorable beliefs about guidelines than physicians, senior clinicians, and subspecialists, respectively.

## Methods

### Overview

Clinicians completed a 27-item survey regarding their beliefs about clinical guidelines and practice variation, as part of a larger study exploring clinical decision-making in the context of written clinical vignettes (manuscript submitted).

### Questionnaire development

An extensive search of PubMed did not reveal an existing questionnaire that specifically addressed our questions regarding clinician attitudes about practice variation, so we created a new questionnaire. We used prior surveys and review articles[19, 20, 40–43] to generate a list of relevant themes. From these we developed 14 Likert-type items regarding clinical guidelines and practice variation generally (response options, 1 = strongly disagree, 6 = strongly agree), and 13 items exploring the impact on practice variation of six specific situational or personal factors and seven potential practice changes (response options, 1 = not at all, 4 = very). Authors DAC and DMD, both of whom have specialized training and extensive experience in survey item development, supervised the development process and examined the wording of each item. We iteratively reviewed and revised all items, including incorporation of findings from pilot-testing by two internal medicine physicians. Table 1 contains the final full text of all items. Respondents self-reported demographic information on their provider type, time in practice, specialty, and practice location. The survey also asked respondents to describe how they would manage four hypothetical patients (written clinical vignettes, not reported in this manuscript) presenting for routine management of hyperlipidemia or syncope. Two of the Likert-type items and all 13 impact items appeared after the vignettes; since not all respondents completed the vignettes, the response rate for these "second half" items is lower than for the 12 "first half" items.

**Table 1:** Main survey results.

Statement or question	Mean (SD), median^a^	No./N (%) agree or moderate/high^a^
**Indicate your level of agreement with the following statements (range 1–6**^a^**)**:		
Clinicians should always apply the latest research findings to each patient.	4.5 (1.0), 5	214/250 (86%)
Clinicians have a responsibility to help reduce the overall cost of medical care.^b^	5.2 (0.8), 5	245/250 (98%)
Reducing variation in clinical practice would benefit most patients.^b^	4.6 (1.1), 5	216/249 (87%)
It is hard to find and quickly comprehend state-of-the-art practice standards when I need them.	3.5 (1.3), 4	130/250 (52%)
Reducing variation in clinical practice would reduce costs.	4.4 (1.1), 5	201/249 (81%)
I trust the findings in most research studies and systematic reviews.	4.2 (0.9), 4	200/248 (81%)
I depend on practice guidelines to help me provide optimal care for my patients.^b^	4.9 (0.9), 5	234/249 (94%)
It is easy to apply practice guidelines to most of my patients.	4.3 (1.0), 4	197/247 (80%)
Most practice variation among clinicians is justified by relevant differences in clinical situations.^b^	3.9 (1.2), 4	161/250 (64%)
I am quick to adapt my clinical practice to align with new practice guidelines.	4.5 (0.9), 5	223/249 (90%)
Clinicians should encourage patients to follow guideline recommendations for diagnosis and treatment.	4.9 (0.8), 5	239/248 (96%)
Clinicians should resist patient requests that are not grounded in solid evidence of benefit.	4.4 (1.1), 4	202/250 (81%)
Between-clinician practice variation *should* be substantially reduced.^c^	4.5 (1.1), 5	133/152 (88%)
Between-clinician practice variation *can* realistically be substantially reduced.^c^	4.1 (1.0), 4	121/153 (79%)
**How much impact do the following factors have on between-clinician variation in practice? (range 1–4**^a^**)**		
Lack of access to needed evidence and guidelines.^c^	2.6 (0.9), 3	87/153 (57%)
Lack of awareness of existing evidence and guidelines.^c^	3.0 (0.7), 3	115/153 (75%)
Differences in practice context and patient population.^c^	3.0 (0.7), 3	118/152 (78%)
Differences in clinician experience and training.^c^	3.2 (0.6), 3	134/153 (88%)
Differences in clinician style and preferences.^c^	3.3 (0.6), 3	136/153 (89%)
Individual patient preferences.^c^	2.9 (0.8), 3	99/153 (65%)
**How helpful would each of the following be in helping you to standardize your clinical practice with other clinicians? (range 1–4**^a^**)**		
Better access to guidelines and synthesized evidence.^c^	3.0 (0.9), 3	107/153 (70%)
More time to look up, appraise, and apply available practice standards.^c^	3.5 (0.7), 4	135/153 (88%)
Clearly stated institution-wide standard practices.^c^	3.1 (0.9), 3	116/153 (76%)
Standardized order sets.^c^	3.1 (0.9), 3	114/153 (75%)
Decision aids to help with patient counseling.^c^	3.2 (0.8), 3	124/153 (81%)
More frequent feedback on how my practice compares with that of others.^c^	3.0 (0.9), 3	103/152 (68%)
Having someone else order common / straightforward tests.^c^	2.3 (1.1), 2	58/153 (38%)

^a^ For the first 14 items the response options were: 1 = strongly disagree, 2 = disagree, 3 = slightly disagree, 4 = slightly agree, 5 = agree, 6 = strongly agree; we dichotomized 4–6 as "agree." For the remaining items the response options were: 1 = not at all, 2 = slight, 3 = moderate, 4 = very; we dichotomized 3–4 as "moderate/high."

^b^ Key item (used to compare respondents who completed the second half of the survey and those who completed only the first half).

^c^ Item from the second half of the survey, which participants completed after they responded to four written clinical vignettes.

### Sampling, participants, and survey administration

We sent email invitations to all 617 clinicians (458 physicians, 123 nurse practitioners, and 36 physician assistants) in the Mayo-Rochester, MN Divisions of General Internal Medicine (N = 123), Primary Care Internal Medicine (N = 86), and Cardiology (N = 228), the Mayo-Rochester Department of Family Medicine (N = 92), and the Mayo Clinic Health System community sites in Austin, MN (N = 19) and La Crosse, WI (N = 69). Participants were offered a monetary incentive. The study was judged exempt by the Mayo Clinic Institutional Review Board.

We used SurveyMonkey to administer the questionnaire from 26 February to 20 April, 2016. The invitation email indicated that this was a "research study to understand how clinicians make decisions at the point of care." All data were anonymous although we did track responses. Invitees received up to two reminders.

Our original goal was to obtain data from 120 participants, and we over-enrolled participants in order to allow for those who might start but not finish the survey. The response to our invitations and the volunteer rate were higher than expected, such that we exceeded our target and had to close the survey before all those invited had a chance to respond, due to limited funds for participant remuneration.

### Statistical analyses

We report means, median, and standard deviation for each item. We determined the frequency of favorable responses (e.g., "agree" or "important") by dichotomizing results as above or below the midpoint of the respective scale. We classified time in practice as early (≤10 years), mid (11–20 years), and late (>20 years) career. We used Kruskal-Wallis tests to compare responses across demographic subgroups. We used Cohen's d to calculate a standardized mean difference for statistically significant models, and classified these differences as small (d = 0.2–0.49), moderate (d = 0.5–0.79), or large (d ≥ 0.8). To compare those who completed the entire survey vs those who completed only the first half we used chi-squared tests to compare demographic features, and used Kruskal-Wallis tests to compare responses for four a priori "key items" (regarding responsibility to reduce cost, benefit of reducing variation, dependence on guidelines, and justification of variation). We used Wilcoxon signed rank tests to compare item responses within individuals. To explore the possibility that nonrespondents were systematically different from respondents we compared available demographic information (provider type, specialty, and practice location) among respondents and non-respondents using the chi-squared test. Because we stopped enrollment before all those invited could respond, we modeled the probability of participation as a function of the time allowed to reply to the survey invitation. From this model, we predicted the probability of participation for all individuals according to the larger of either 14 days (the per-protocol minimum) or the actual time allowed to respond to the invitation. The "expected" response rate was the average of these individual-level predictions that accounted for the early withdrawal of some invitations. Given the large number of comparisons, we used a two-tailed alpha level of 0.01 as the threshold of statistical significance. We used SAS version 9.4 (SAS Institute Inc., Cary, North Carolina). We powered the study for the analysis of the vignette management (to be reported elsewhere), and estimated the required sample size at 120. We did not separately power this survey study.

## Results

Two hundred fifty clinicians completed at least one questionnaire item (response rate 41%). We found no statistically significant differences in provider type, specialty, or practice location for respondents vs nonrespondents. As a secondary estimate of response, we determined the response rate expected if all those invited had had an equal chance of responding (i.e., if none had been uninvited) at 45%.

The sample included 178 physicians, 60 nurse practitioners, and 12 physician assistants. Respondents had been in clinical practice a mean (SD) of 15.1 (11.3) years. Table 2 lists additional demographics. We found no statistically significant differences between the 153 respondents (61%) who completed the second half of the survey and those who completed only the first half, in demographic features (provider type, time in practice, specialty, or practice location; p>.15) or in responses to the key questionnaire items (listed in Methods; p>.06).

**Table 2:** Characteristics of invitees and respondents.

Demographic	Feature	Invited No. (%)	Respondents No. (%)
**Provider type**	Physician	458 (74%)	178 (71%)
	Nurse practitioner	123 (20%)	60 (24%)
	Physician assistant	36 (6%)	12 (5%)
**Years in practice**	≤10	--	109 (45%)
	11–20	--	66 (27%)
	>20	--	69 (28%)
**Specialty**	Cardiology	227 (37%)	95 (38%)
	Family medicine	181 (29%)	76 (30%)
	Internal medicine	209 (34%)	79 (32%)
**Practice location**	Academic	528 (86%)	213 (85%)
	Community	89 (14%)	37 (15%)

N = 250 respondents. Years in practice information not available for invited cohort, and not reported by 6 respondents.

### Main survey findings

#### Impact and sources of variation in care

Clinicians generally agreed that practice variation *should* be reduced (mean [standard deviation], 4.5 [1.1]; 1 = strongly disagree, 6 = strongly agree), but 65/152 (43%) respondents agreed less strongly that it *can* realistically be reduced (4.1 [1.0], p < .001). Clinicians believed that reducing variation would benefit most patients (4.6 [1.1]), and only slightly agreed that variation is justified by differences in clinical situations (3.9 [1.2]).

When asked to rate factors that influence practice variation (1 = no impact, 4 = high impact), respondents perceived differences in clinician style (3.3 [0.6]) and experience (3.2 [0.6]) as most influential, while patient differences (3.0 [0.7]) and preferences (2.9 [0.8]) were moderately influential. Lack of access to guidelines was only slightly/moderately influential (2.6 [0.9]).

When asked to rate factors that might help to standardized practice (1 = not at all helpful, 4 = very helpful), respondents rated time to identify and apply standards the highest (3.5 [0.7]), followed by patient decision aids (3.2 [0.8]), institution-wide standard practices (3.1 [0.9]), and standardized order sets (3.1 [0.9]). Having someone else order common tests was rated lowest (2.3 [1.1]).

#### Cost

Clinicians strongly agreed (5.2 [0.8], range 1–6) that clinicians have a responsibility to reduce the cost of care. They agreed less strongly (4.4 [1.1]) that reducing practice variation would reduce costs.

#### Guidelines and evidence

Nearly all respondents (234/249 [94%]) agreed that they depend on practice guidelines to help them provide patient care (mean 4.9 [0.9], range 1–6). They reported that they are quick to adapt their own practice to align with new guidelines (4.5 [0.9]) and agreed that clinicians should apply new research findings (4.5 [1.0]). Repondents were slightly less favorable regarding the ease of applying guidelines to their patients (4.3 [1.0]) and their trust of research (4.2 [0.9]). Only 130/250 (52%) agreed that practice standards are hard to find and apply (mean 3.5 [1.3]).

Nearly all (239/248 [96%]) agreed that clinicians should encourage patients to follow guideline recommendations (4.9 [0.8]). The also agreed, but less strongly, that clinicians should resist patient requests that are not evidence-based (4.4 [1.1]).

### Analyses across clinician demographic subgroups

Differences across provider type, time in practice, specialty, and practice location were generally small and not statistically significant (see Table 3, and S1 eTable). The statistically significant (at alpha 0.01) comparisons across provider type were that nurse practitioners and physician assistants rated more highly than physicians their trust in research (Cohen's d = 0.42), dependence on practice guidelines (d = 0.35), quickness to align with practice guidelines (d = 0.32), and five actions that might help standardize their clinical practice (better access to guidelines and evidence, d = 0.65; more time to look up, appraise, and apply standards, d = 0.70; institution-wide standard practices, d = 0.87; decision aids for patient counseling, d = 0.56; and practice feedback, d = 0.47); all p≤.008.

**Table 3:** Survey results: Subgroup analyses by provider type and years in practice.

	Provider type			Years in practice			
Question / statement	Physicians N = 178 Mean (SD)	NP/PA N = 72 Mean (SD)	P	≤10 yrs N = 109 Mean (SD)	11–20 yrs N = 66 Mean (SD)	>20 yrs N = 69 Mean (SD)	P
**Agree / disagree (range 1–6)**							
Apply latest research findings	4.4 (1.0)	4.6 (1.1)	.08	4.6 (1.0)	4.5 (1.0)	4.2 (1.2)	.12
Responsibility to reduce costs	5.2 (0.8)	5.1 (0.8)	.30	5.2 (0.8)	5.3 (0.7)	5.1 (0.9)	.81
Reducing variation would benefit patients^a^	4.6 (1.0)	4.4 (1.2)	.68	4.6 (1.1)	4.6 (1.1)	4.5 (1.2)	.96
Hard to find & understand practice standards	3.5 (1.3)	3.3 (1.3)	.26	3.7 (1.3)	3.3 (1.3)	3.2 (1.2)	.02
Reducing variation would reduce costs^a^	4.4 (1.1)	4.3 (1.1)	.30	4.4 (1.0)	4.4 (1.1)	4.4 (1.3)	.62
Trust research & systematic reviews^c^	4.1 (0.9)	4.5 (1.0)	.001	4.2 (1.0)	4.2 (0.9)	4.1 (0.9)	.74
Depend on practice guidelines^a^	4.8 (0.9)	5.1 (0.9)	.002	5.0 (0.9)	5.0 (0.8)	4.6 (0.9)	.004
Easy to apply practice guidelines^d^	4.2 (1.0)	4.5 (1.0)	.02	4.4 (1.0)	4.3 (1.0)	4.1 (1.0)	.31
Variation justified by clinical situations	3.8 (1.2)	4.2 (1.1)	.03	4.1 (1.1)	4.0 (1.2)	3.6 (1.2)	.03
Quick to adapt to align with guidelines^a^	4.4 (0.8)	4.7 (0.9)	.008	4.6 (0.9)	4.4 (0.8)	4.4 (0.9)	.22
Encourage patients to follow guideline recommendations^c^	4.8 (0.7)	4.9 (0.8)	.18	4.9 (0.7)	4.8 (0.8)	4.8 (0.8)	.48
Resist patient requests not grounded in evidence	4.4 (1.0)	4.3 (1.2)	.91	4.4 (1.0)	4.3 (1.2)	4.3 (1.1)	.74
Variation *should* be reduced^a^^,^^b^	4.5 (1.1)	4.6 (0.9)	.64	4.5 (1.1)	4.7 (1.0)	4.4 (1.1)	.32
Variation *can* realistically be reduced^b^	4.1 (1.0)	4.0 (0.9)	.44	4.1 (0.8)	4.2 (1.2)	3.9 (0.9)	.25
**How much impact on between-clinician variation? (range 1–4)**							
Lack access to evidence & guidelines^b^	2.6 (0.8)	2.8 (0.9)	.12	2.6 (0.8)	2.6 (0.9)	2.6 (0.9)	.91
Lack awareness evidence & guidelines^b^	2.9 (0.7)	3.0 (0.7)	.39	3.0 (0.7)	3.1 (0.9)	2.8 (0.7)	.16
Differences in context & patient population^a^^,^^b^	3.0 (0.7)	3.0 (0.7)	.90	3.1 (0.7)	3.0 (0.6)	2.9 (0.7)	.53
Differences in clinician experience & training^b^	3.2 (0.7)	3.3 (0.6)	.22	3.4 (0.6)	3.1 (0.7)	3.1 (0.6)	.04
Differences in clinician style & preferences^b^	3.2 (0.7)	3.3 (0.6)	.75	3.2 (0.6)	3.3 (0.7)	3.2 (0.7)	.72
Individual patient preferences^b^	2.8 (0.8)	3.0 (0.8)	.08	2.8 (0.8)	2.9 (0.9)	2.9 (0.7)	.89
**How helpful to standardize your practice? (range 1–4)**							
Access to guidelines & evidence^b^	2.8 (0.9)	3.4 (0.8)	< .001	3.1 (0.9)	3.1 (0.9)	2.7 (1.0)	.11
Time to look up, appraise, & apply standards^b^	3.3 (0.7)	3.8 (0.5)	< .001	3.6 (0.7)	3.5 (0.7)	3.1 (0.8)	< .001
Clear institution-wide standard practices^b^	2.9 (0.9)	3.6 (0.6)	< .001	3.3 (0.7)	3.1 (1.0)	2.7 (0.9)	.008
Standardized order sets^b^	3.0 (0.9)	3.3 (0.9)	.07	3.3 (0.8)	3.1 (1.0)	2.9 (0.9)	.12
Decision aids for patient counseling^b^	3.1 (0.9)	3.6 (0.6)	.001	3.4 (0.8)	3.4 (0.8)	2.9 (0.9)	.005
Feedback comparing my practice with others^a^^,^^b^	2.8 (1.0)	3.3 (0.8)	.009	3.0 (0.9)	3.1 (1.0)	2.6 (0.9)	.03
Someone else order straightforward tests^b^	2.2 (1.1)	2.3 (1.1)	.62	2.2 (1.1)	2.2 (1.1)	2.4 (1.0)	.71

NP/PA = nurse practitioner / physician assistant, SD = standard deviation, yrs = years. See Table 1 for exact wording and response options for each item. Six respondents did not report time in practice.

^a^ Missing 1 data point (N = item total-1).

^b^ Sample size N = 153 for this item unless otherwise noted by additional footnote; provider type N = 105 physicians, N = 48 NP/PAs; time in practice N = 70 at ≤10 years, N = 39 at 11–20 years, N = 39 at >20 years.

^c^ Missing 2 data points (N = item total-2).

^d^ Missing 3 data points (N = item total-3).

We found statistically significant differences by time in practice for four items. Early- or mid-career clinicians reported higher agreement than late-career clinicians in dependence on practice guidelines (d = 0.42, p = .01); and rated time, institution-wide standard practices, and decision aids as more helpful in standardizing practice (d = 0.47–0.60, p≤.006).

Only one analysis by specialty reached statistical significance, namely that cardiologists agreed more than internal medicine or family medicine clinicians that practice variation should be reduced (d = 0.29, p = .01; see S1 eTable). None of the comparisons between academic vs community practice location were statistically significant (p>.01; see S1 eTable).

## Discussion

The physicians, nurse practitioners, and physician assistants responding to this survey agreed that practice variation should be reduced, but many seemed to have reservations about the feasibility of this aim. Time to appraise and apply practice standards was rated as most helpful in standardizing practice. By contrast, access to guidelines was not perceived to be an important factor in creating or resolving practice variation. Clinicians perceived that differences in clinician style and experience have more influence on variation than patient differences or preferences. In subgroup analyses, nurse practitioners and physician assistants had more favorable beliefs than physicians about practice guidelines and the potential value of several other changes to help standardize practice. Younger clinicians rated nearly all proposed approaches to standardization as more helpful. These differences were, with one exception, small or moderate in magnitude (Cohen's d<0.8). Generalists and specialists, and those at academic vs community sites, had overall similar beliefs.

### Limitations

The generalizability of our findings is limited by the response rate and the sampling from a single geographic region and health system. While it is possible that those completing the survey had stronger feelings about the topic than those who did not, the invitation email did not mention practice variation or clinical practice guidelines, and respondents were similar to nonrespondents for available demographics. We acknowledge the large number of statistical tests and suggest that statistically significant findings should be interpreted cautiously, highlighting potentially interesting relationships that may merit further study. Finally, it is possible that those responding to the second-half items were systematically different from those who responded only to the first-half items (although we found no differences between these groups in demographic features or responses to the key items), or that that the clinical vignettes could have shaped their responses to the second-half items.

Strengths include the sample that encompassed multiple provider types and specialties, and both academic and community sites; the carefully developed survey; and the sample size that exceeded our planned enrollment.

### Integration with prior work

Our findings extend those of earlier qualitative studies[35, 36, 40] by prioritizing the factors that influence variation. Our findings also parallel prior work exploring the adoption of or resistance to clinical practice guidelines, namely that physicians have favorable attitudes about practice guidelines in general yet remain somewhat skeptical of specific recommendations, have difficulty applying them to specific patients, and lack time to do so.[18–20] A recent review of studies exploring non-adherence to guidelines found that much non-adherence is intentional, and at least partly warranted by patient decisions or case-specific contraindications.[13] Similar to our results, at least one US study has found more favorable attitudes among younger physicians.[44] By contrast with our findings, a study in Norway found significantly greater challenges for generalists compared with non-generalist physicians.[43]

### Implications

The clinicians in this sample did not express strong concerns about practice variation: about two-thirds agreed that variation is justified by relevant clinical differences, and the vast majority indicated that they already follow guidelines themselves. While some variation is indeed justified,[13] studies suggest that non-adherence to guidelines is widespread.[1, 16, 45] Moreover, clinicians' perceptions of their own guideline adherence is typically more favorable than reality.[46–48] Yet even if clinicians are mistaken in their beliefs, their perceptions must be taken seriously and at face value. It will be difficult to fix the problem if those involved fail to recognize its magnitude, importance, or potential for correction. Changing attitudes will require that beliefs be acknowledged, needs understood, evidence sought, and misperceptions corrected. Systems-level solutions will play an essential role in standardizing practice, but attempts to circumvent clinicians, i.e., through automated implementation of guidelines, may backfire. We note, for example, that the lowest-rated item on our survey was a potential practice change in which someone else would order common or straightforward tests.

Physicians seem to have lingering doubts that practice variation can be reduced. This may reflect low "outcome expectancy"–the belief that certain outcomes will result from given actions.[49] Before clinicians will invest personal effort, they must believe that their efforts will result in desired outcomes.[18] Low outcome expectancy may reflect concerns about the effectiveness of currently-available solutions including guidelines,[18, 19, 21, 22] decision aids,[25] clinical decision support tools,[27] and individual performance feedback.[30] Research to identify new solutions, and how to more effectively implement existing ideas, remains a top priority. Alternatively, doubts about reducing practice variation may reflect a lack of personal self-efficacy—the capability to perform at a certain level in a specific task and context.[50] Research exploring how to influence clinicians' self-efficacy and other motivations may prove insightful.[49, 51, 52]

Although younger clinicians and nurse practitioners and physician assistants rated several items higher than senior clinicians and physicians, relative prioritizations (rankings) were generally uniform across subgroups. This suggests that solutions need not target specific groups during development, although testing during implementation would be warranted.

Perhaps most importantly, our respondents indicated that the factor anticipated to most help standardize their clinical practice is not new standards or better access to guidelines, but is rather *time* to apply existing standards. Others have reported similar beliefs.[18, 19] This suggests that instead of focusing attention exclusively on developing and implementing guidelines, we might have more success by freeing up clinicians' time or reducing the time needed to apply patient-tailored guideline recommendations. Since decreasing clinicians' time burden is likely unrealistic (and, even if achieved, might not actually be used to study and apply standards), we propose to focus on how to efficiently integrate practice standards into clinicians' workflow.

## Conclusions

Clinicians believe that practice variation should be reduced, but are less certain that this can be achieved. Accessibility of guidelines is not a significant barrier to practice standardization, whereas more time to apply standards is viewed as potentially helpful.

## Supporting information

S1 eTable Survey results: Subgroup analyses by specialty and practice location. (DOCX)
