# Supplementary material for: Practice variation and practice guidelines: Attitudes of generalist and specialist physicians, nurse practitioners, and physician assistants
Source: PLoS One. 2018 Jan 31;13(1):e0191943. doi: 10.1371/journal.pone.0191943 (PMC5792011; doi:10.1371/journal.pone.0191943)
Supplement: S1 eTable — (DOCX) [file pone.0191943.s001.docx]

# eTable. Survey results: Subgroup analyses by specialty and practice location

|  | **Specialty^a^**  **Mean (SD)** | | | **Practice location^a^**  **Mean (SD)** | |
| --- | --- | --- | --- | --- | --- |
| **Statement or question** | **Family Medicine**  **N=76** | **Internal Medicine**  **N=79** | **Cardiology**  **N=95** | **Academic**  **N=213** | **Community**  **N=37** |
| **Agree / disagree (range 1-6)** |  |  |  |  |  |
| Clinicians should always apply the latest research findings to each patient. | 4.6 (1.0) | 4.3 (1.1) | 4.5 (1.0) | 4.4 (1.1) | 4.7 (0.8) |
| Clinicians have a responsibility to help reduce the overall cost of medical care. | 5.3 (0.7) | 5.0 (0.9) | 5.3 (0.7) | 5.2 (0.8) | 5.4 (0.6) |
| Reducing variation in clinical practice would benefit most patients.^b^ | 4.5 (0.9) | 4.4 (1.1) | 4.7 (1.1) | 4.5 (1.1) | 4.6 (1.0) |
| It is hard to find and quickly comprehend state-of-the-art practice standards when I need them. | 3.2 (1.2) | 3.5 (1.2) | 3.6 (1.3) | 3.5 (1.3) | 3.6 (1.3) |
| Reducing variation in clinical practice would reduce costs.^b^ | 4.3 (1.1) | 4.3 (1.1) | 4.5 (1.1) | 4.4 (1.1) | 4.2 (1.3) |
| I trust the findings in most research studies and systematic reviews.^d^ | 4.3 (0.9) | 4.3 (0.8) | 4.0 (1.0) | 4.2 (0.9) | 4.2 (0.9) |
| I depend on practice guidelines to help me provide optimal care for my patients.^b^ | 4.8 (1.1) | 4.8 (0.8) | 5.0 (0.8) | 4.9 (0.9) | 4.8 (1.1) |
| It is easy to apply practice guidelines to most of my patients.^e^ | 4.4 (1.1) | 4.1 (1.1) | 4.3 (0.9) | 4.3 (1.0) | 4.1 (1.3) |
| Most practice variation among clinicians is justified by relevant differences in clinical situations. | 4.0 (1.2) | 3.9 (1.0) | 3.9 (1.3) | 3.9 (1.2) | 4.0 (1.3) |
| I am quick to adapt my clinical practice to align with new practice guidelines.^b^ | 4.4 (0.9) | 4.4 (0.9) | 4.7 (0.8) | 4.5 (0.8) | 4.5 (1.0) |
| Clinicians should encourage patients to follow guideline recommendations for diagnosis and treatment.^a,d^ | 4.9 (0.7) | 4.7 (0.8) | 5.0 (0.7) | 4.9 (0.7) | 4.8 (0.8) |
| Clinicians should resist patient requests that are not grounded in solid evidence of benefit. | 4.2 (0.9) | 4.4 (1.0) | 4.4 (1.2) | 4.4 (1.1) | 4.0 (0.9) |
| Between-clinician practice variation *should* be substantially reduced.^b,c^ | 4.4 (1.1) | 4.3 (0.8) | 4.8 (1.1) | 4.5 (1.0) | 4.5 (1.3) |
| Between-clinician practice variation *can* realistically be substantially reduced.^c^ | 3.9 (1.0) | 4.0 (0.8) | 4.3 (1.0) | 4.1 (0.9) | 4.1 (1.1) |
| **How much impact on between-clinician variation? (range 1-4)** |  |  |  |  |  |
| Lack of access to needed evidence and guidelines.^c^ | 2.5 (0.8) | 2.7 (0.8) | 2.7 (1.0) | 2.6 (0.9) | 2.5 (0.7) |
| Lack of awareness of existing evidence and guidelines.^c^ | 2.9 (0.7) | 3.0 (0.7) | 3.0 (0.8) | 3.0 (0.7) | 2.9 (0.7) |
| Differences in practice context and patient population.^b,c^ | 2.9 (0.7) | 3.1 (0.7) | 3.0 (0.7) | 3.1 (0.7) | 2.8 (0.7) |
| Differences in clinician experience and training.^c^ | 3.2 (0.6) | 3.3 (0.7) | 3.2 (0.7) | 3.2 (0.7) | 3.3 (0.6) |
| Differences in clinician style and preferences.^c^ | 3.3 (0.6) | 3.1 (0.7) | 3.3 (0.7) | 3.2 (0.7) | 3.4 (0.5) |
| Individual patient preferences.^c^ | 3.0 (0.7) | 2.9 (0.7) | 2.6 (0.8) | 2.8 (0.8) | 3.1 (0.8) |
| **How helpful to standardize your practice? (range 1-4)** |  |  |  |  |  |
| Better access to guidelines and synthesized evidence.^c^ | 3.0 (0.9) | 3.0 (0.8) | 3.1 (1.0) | 3.0 (0.9) | 3.0 (1.0) |
| More time to look up, appraise, and apply available practice standards.^c^ | 3.5 (0.7) | 3.4 (0.7) | 3.4 (0.7) | 3.4 (0.7) | 3.5 (0.7) |
| Clearly stated institution-wide standard practices.^c^ | 3.2 (0.9) | 3.0 (0.8) | 3.2 (0.9) | 3.1 (0.9) | 3.0 (0.9) |
| Standardized order sets.^c^ | 3.2 (0.9) | 3.1 (0.8) | 3.1 (1.0) | 3.1 (0.9) | 3.2 (0.9) |
| Decision aids to help with patient counseling.^c^ | 3.2 (0.9) | 3.1 (0.8) | 3.4 (0.8) | 3.2 (0.8) | 3.2 (0.9) |
| More frequent feedback on how my practice compares with that of others.^b,c^ | 2.9 (1.0) | 3.0 (0.8) | 3.0 (0.9) | 3.0 (0.9) | 2.8 (1.1) |
| Having someone else order common / straightforward tests.^c^ | 2.4 (1.1) | 2.4 (1.2) | 2.0 (0.9) | 2.3 (1.0) | 2.2 (1.1) |

See main text Table 1 for exact wording and response options for each item. SD = standard deviation.

^a^ P>.01 for all comparisons except p=.01 for comparison across specialties for "Between-clinician practice variation should be substantially reduced."

^b^ Missing 1 data point (N=item total-1).

^c^ Sample size N=153 for this item unless otherwise noted by additional footnote; N=51 for each specialty, N=129 academic, N=24 community.

^d^ Missing 2 data points (N=item total-2).

^e^ Missing 3 data points (N=item total-3).
